# Nitrogen Oxide Inhalation Lung Injury From an Industrial Explosion: A Case Report and Review of the Literature

**DOI:** 10.7759/cureus.93491

**Published:** 2025-09-29

**Authors:** Hitokazu Tsukao

**Affiliations:** 1 Department of Respiratory Internal Medicine, Fukui Prefectural Hospital, Fukui, JPN

**Keywords:** accidents, industrial, lung injuries, nitrogen oxides, pathology, radiology

## Abstract

An industrial explosion released nitrogen oxide, which a 43-year-old man inhaled at work. He developed cough and exertional dyspnea without hypoxemia, and chest computed tomography obtained two days after exposure showed poorly defined centrilobular ground-glass nodules predominantly in the lingular and lower lobes. Bronchoalveolar lavage revealed an increased total cell count with mild lymphocytosis. Transbronchial lung biopsy demonstrated type II pneumocyte hyperplasia, mural thickening with focal fibrosis, and fibrin deposition consistent with centri-acinar dominant alveolitis. Supportive care without corticosteroids led to clinical and radiologic resolution.

This case highlights the latent onset pattern after nitrogen oxide inhalation and provides clinicoradiologic-pathologic correlation for a mild presentation, suggesting that careful observation may be reasonable when oxygenation is preserved and no progression is observed.

## Introduction

Nitrogen oxides (NOx) are toxic gases generated during nitric acid decomposition, industrial explosions or fires, and metal surface treatment or oxidation processes. In agriculture, NOx exposure has long been recognized as “silo-filler’s disease” [1,2].

Inhalation typically causes cough, dyspnea, and fever after a latent period of several hours up to 72 hours and may progress to acute respiratory distress syndrome (ARDS) in severe cases [3,4]. At the pathophysiological level, NOx generates reactive nitrogen species that damage the alveolar epithelium and capillary endothelium, leading to increased permeability and inflammation. Late complications, including bronchiolitis obliterans, organizing pneumonia, or pulmonary fibrosis, have also been reported [5].

Exposure scenarios are diverse, including industrial explosions, tank-cleaning accidents, and chemical reaction incidents [6-8]. More recently, a single severe case managed with veno-venous extracorporeal membrane oxygenation (VV-ECMO) has also been reported [9]. Radiologically, poorly defined centrilobular nodules and ground-glass opacities are characteristic findings [10].

Most previously reported cases were moderate-to-severe, and fatalities are not uncommon [11]. Corticosteroids are often administered [12], but consensus regarding optimal dose or duration is lacking. Furthermore, therapeutic strategies for mild cases remain uncertain, and spontaneous improvement without corticosteroid therapy has been observed [13,14].

Reports that correlate a mild clinical course with pathological findings are scarce. We present a case of mild NOx inhalation injury following an industrial explosion, in which we were able to demonstrate concordant radiologic and histopathologic features. This case may provide new insights into the diagnosis and management of mild NOx lung injury.

## Case presentation

A 43-year-old male chemical plant worker, a non-smoker with no passive smoking exposure, presented after an industrial explosion in July 2018. At the time of the accident, a chemical reaction involving 67.5% nitric acid, 4-t-butylcyclohexanol, and ammonium metavanadate was ongoing. An orange-colored gas cloud was documented in on-site photographs. Without respiratory protection, he entered the site and developed malaise. Approximately three hours later, he was transported to the emergency department. Initial chest CT showed no abnormalities, and he was discharged home.

**Figure 1:**
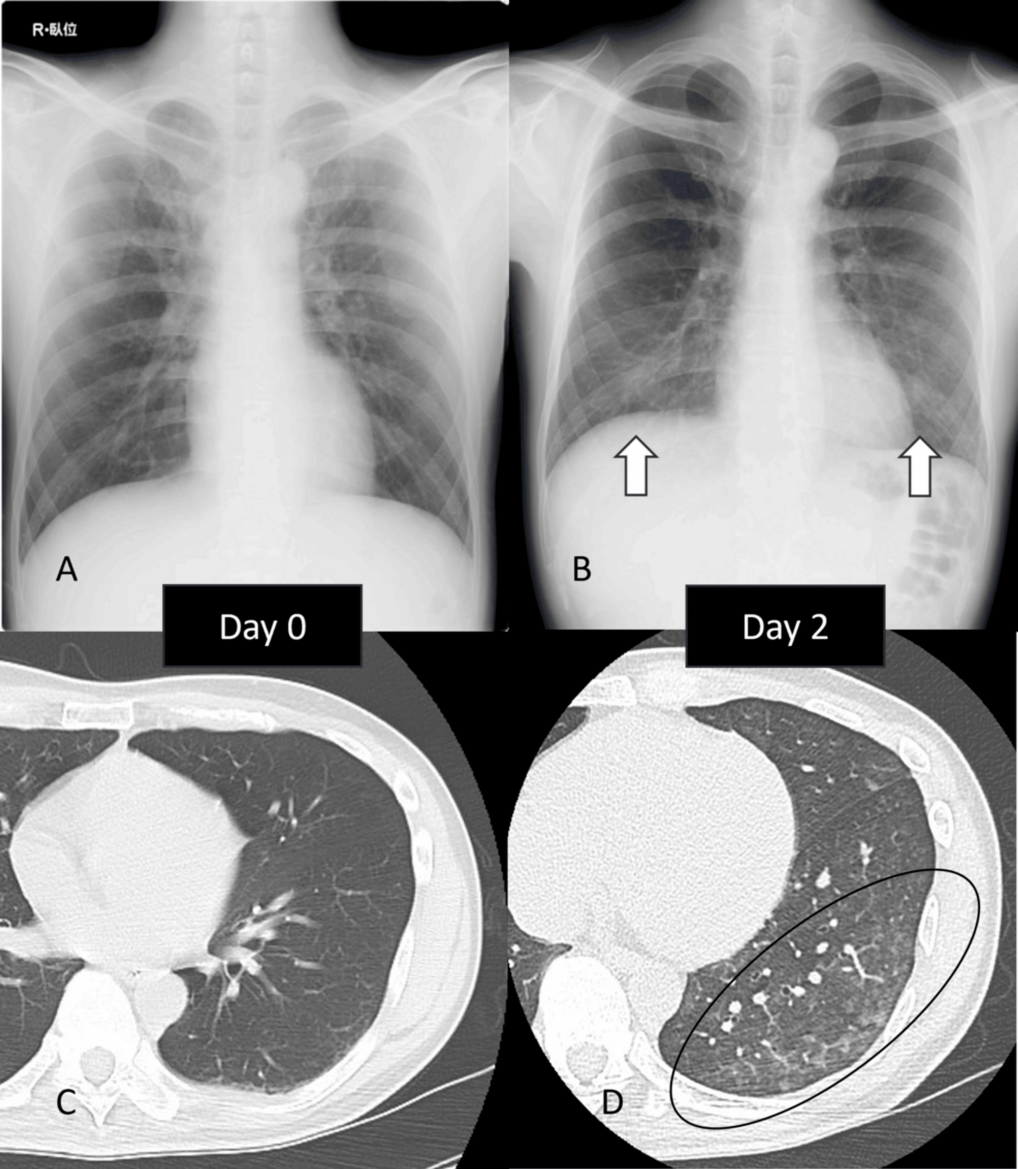
Radiographic and HRCT findings of nitrogen oxide inhalation injury (A) Chest radiograph on Day 0 showing no abnormalities. (B) Chest radiograph on Day 2 demonstrating new patchy bilateral opacities (arrows). (C) HRCT on Day 0 showing normal lung fields. (D) HRCT on Day 2 revealing poorly defined centrilobular ground-glass nodules in the lingula and bilateral lower lobes (circle). HRCT: high-resolution computed tomography

On days 1-2 (18-42 hours after exposure), cough and dyspnea worsened. Chest radiography revealed patchy bilateral opacities, and high-resolution computed tomography (HRCT) demonstrated poorly defined centrilobular ground-glass nodules in the lingular and lower lobes (Figure 1).

On admission, vital signs were stable (peripheral oxygen saturation (SpO₂) 98% on room air). Laboratory testing revealed leukocytosis with a left shift, elevated C-reactive protein (CRP), and mild increases in lactate dehydrogenase (LDH) and creatine phosphokinase (CPK), while angiotensin-converting enzyme (ACE) and soluble interleukin-2 receptor (sIL-2R) levels were within normal limits (Table 1).

**Table 1:** Laboratory and bronchoalveolar lavage findings on admission Reference ranges are shown for each parameter. ACE, Angiotensin-converting enzyme; ALP, Alkaline phosphatase; ALT, Alanine aminotransferase; AST, Aspartate aminotransferase; BAL, Bronchoalveolar lavage; BNP, Brain natriuretic peptide; BUN, Blood urea nitrogen; CPK, Creatine phosphokinase; CRP, C-reactive protein; FCOHb, Fractional carboxyhemoglobin; FiO₂, Fraction of inspired oxygen; γ-GTP, Gamma-glutamyl transpeptidase; Hb, Hemoglobin; Hct, Hematocrit; KL-6, Krebs von den Lungen-6; LDH, Lactate dehydrogenase; Na, Sodium; K, Potassium; Plt, Platelet count; RBC, Red blood cell count; sIL-2R, Soluble interleukin-2 receptor; SpO₂, Peripheral oxygen saturation; TP, Total protein; WBC, White blood cell count

Parameter	Result	Reference range
WBC	11,690 /µl	3,500–9,000 /µl
Neutrophils (%)	77.1 %	40–70 %
Eosinophils (%)	1.8 %	1–6 %
Basophils (%)	0.3 %	0–1 %
Monocytes (%)	8.1 %	2–8 %
Lymphocytes (%)	12.7 %	20–45 %
RBC	4.78 ×10^6 /µl	4.10–5.30 ×10^6 /µl (M)
Hemoglobin (Hb)	14.8 g/dl	13.5–17.5 g/dl (M)
Hematocrit (Hct)	NA	40–50 % (M)
Platelets (Plt)	28.0 ×10^4 /µl	150–350 ×10^3 /µl
D-dimer	0.20 µg/l	<0.5 µg/ml
FiO₂	21 %	21 % (room air)
pH	Error (unknown)	7.35–7.45
pCO₂	34.2 mmHg	35–45 mmHg
pO₂	91.9 mmHg	80–100 mmHg
Lactate	7.0 mg/dl	4.5–19.8 mg/dl (0.5–2.2 mmol/L)
FCOHb	0.9 %	<2 %
TP	6.5 g/dl	6.6–8.1 g/dl
Albumin	3.3 g/dl	4.1–5.1 g/dl
BUN	0.62 mg/dl	8–20 mg/dl
Creatinine	15.4 mg/dl	0.65–1.07 mg/dl (M)
Na	139 mEq/L	138–145 mEq/L
K	4.3 mEq/L	3.6–4.8 mEq/L
T-Bil	1.3 mg/dl	0.2–1.2 mg/dl
ALP	195 IU/L	106–322 IU/L
AST	17 IU/L	13–33 IU/L
ALT	16 IU/L	8–42 IU/L
LDH	218 IU/L	124–222 IU/L
γ-GTP	14 IU/L	13–64 IU/L
CPK	275 IU/L	45–163 IU/L
BNP	<5.8 pg/ml	<18.4 pg/ml
CRP	1.80 mg/dl	<0.3 mg/dl
KL-6	164.2 U/ml	<500 U/ml
SP-A	32.1 ng/ml	<43.8 ng/ml
SP-D	65.5 ng/ml	<110 ng/ml
ACE	7.0 U/L (37℃)	8.3–21.4 U/L
sIL-2R	348.3 U/ml	122–496 U/ml
BAL Recovery	106/150 ml	≥30 %
BAL TCC	5.12 ×10^5 /ml	1–2 ×10^5 /ml
BAL Neutrophils (%)	6.0 %	<3 %
BAL Eosinophils (%)	7.0 %	<1 %
BAL Lymphocytes (%)	36.0 %	10–15 %
BAL Macrophages (%)	51.0 %	85–95 %
BAL CD4/CD8 ratio	3.04	1.0–3.5
BAL Cytology	Class I	Class I (normal)
BAL Culture	Normal flora	—

**Figure 2:**
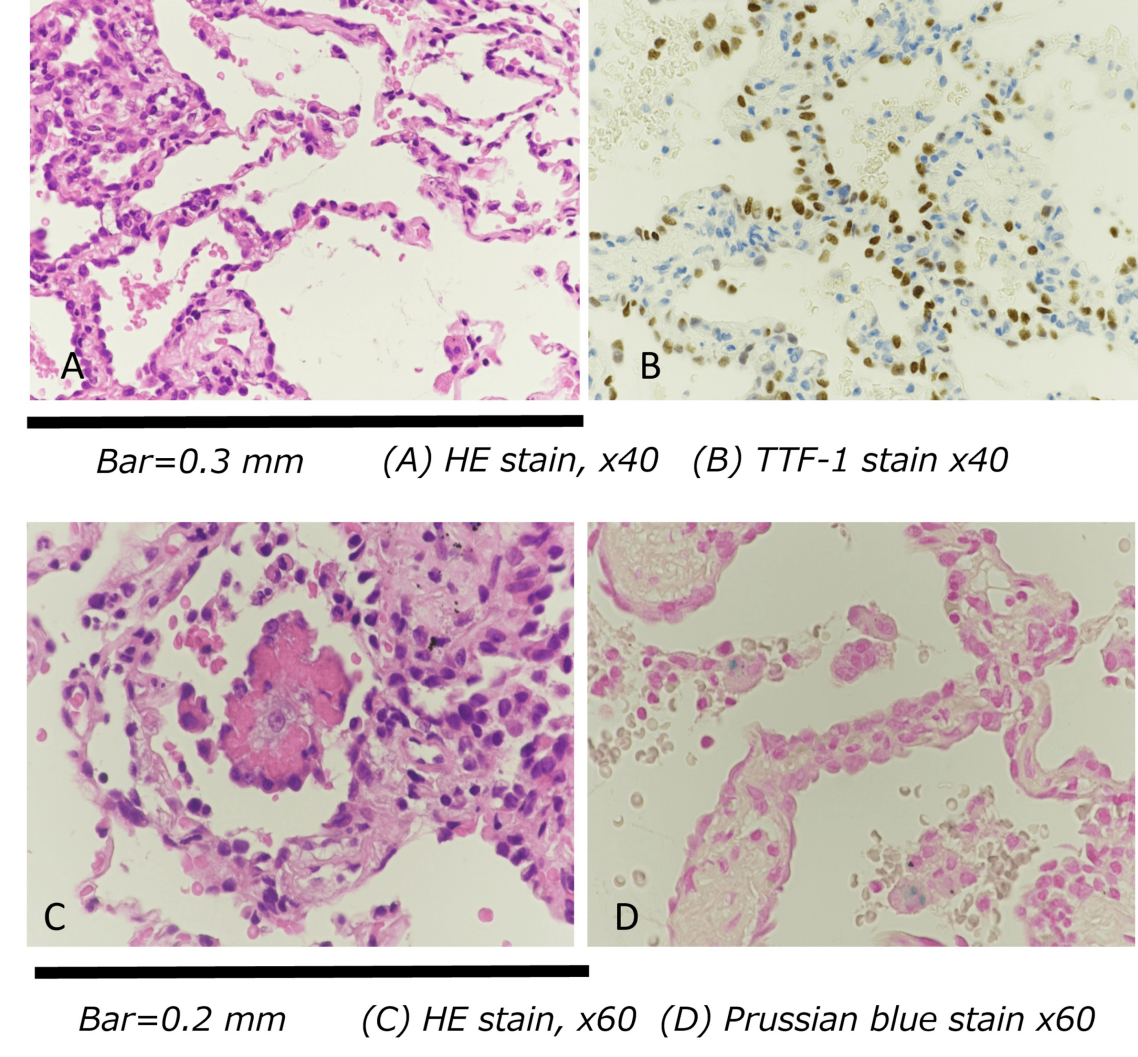
Histopathological findings from transbronchial lung biopsy (A, C) Alveolar septal thickening with type II pneumocyte hyperplasia adjacent to a muscular pulmonary arteriole (hematoxylin and eosin stain, ×200). (B) Thyroid transcription factor-1 (TTF-1) staining positive in hyperplastic alveolar epithelial cells. (D) Intra-alveolar eosinophilic material consistent with fibrin deposition and early organization.

Bronchoscopy revealed no endobronchial abnormalities. Bronchoalveolar lavage fluid was turbid and yellowish, with an increased cell count but negative cultures. Transbronchial lung biopsy demonstrated type II pneumocyte hyperplasia and alveolar wall thickening with fibrosis. Lesions were predominantly distributed around muscular pulmonary arteries, suggesting an airway-centered pattern. Intra-alveolar eosinophilic material consistent with fibrin deposition and early organization was also identified. Collectively, these findings supported a diagnosis of centriacinar-dominant mural alveolitis with focal fibrin deposition (Figure 2).

**Figure 3:**
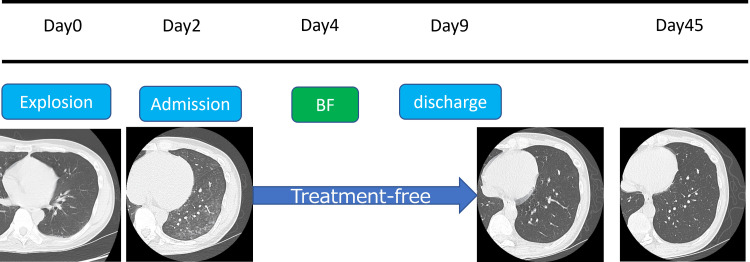
Clinical course Follow-up CT on day 45 after exposure demonstrating resolution of the previously noted centrilobular ground-glass opacities.

The patient was managed supportively without corticosteroids. Oxygenation remained stable throughout hospitalization. Radiographic opacities gradually improved, and he was discharged on day 9. He subsequently developed transient depressive symptoms, which improved with psychiatric intervention. These psychiatric manifestations were interpreted as stress-related reactions to the industrial accident rather than direct toxic effects of NOx exposure. Follow-up CT scans on days 15 and 45 showed marked resolution (Figure 3).

## Discussion

Inhalational injuries may be caused by gases such as chlorine, phosgene, sulfur dioxide, hydrogen sulfide, NOx, ozone, and ammonia [15]. Highly water-soluble gases (chlorine, ammonia, sulfur dioxide) tend to dissolve in the upper airway, whereas poorly soluble gases (phosgene, NOx, ozone) reach the lower respiratory tract and exert delayed effects [16]. Accordingly, highly soluble gases cause acute upper-airway symptoms immediately, while poorly soluble gases cause delayed onset symptoms after a latent period. In our patient, initial imaging was normal, but infiltrates appeared on day 2, consistent with the typical pathophysiology of NOx injury.

The most severe complication is ARDS, usually developing within 24 hours of high-concentration exposure [3]. Late complications, such as organizing pneumonia, bronchiolitis obliterans, and pulmonary fibrosis, have also been documented [5,13]. Thus, attention is required not only in the acute phase but also during long-term follow-up.

Previous reviews have summarized English-language cases, but numerous cases have also been reported in Japanese literature [12]. To contextualize our case within the broader spectrum of NOx inhalation injury, we reviewed 64 cases reported between 1978 and 2018, encompassing both international and domestic reports while excluding duplicates and abstracts (Table 2) [17-26].

**Table 2:** Reported cases of NOx inhalation injury, 1978–2018 This table summarizes reported cases of nitrogen oxide (NOx) inhalation injury published in the English- and Japanese-language literature. Twenty-nine references were included; duplicates and abstracts were excluded. A severe case was defined as PaO₂ < 60 mmHg on room air or the requirement for supplemental oxygen at the initial assessment. Rows marked with an asterisk (*) indicate cases cited from secondary Japanese sources because the original pre-Internet articles were inaccessible. Secondary sources used for data abstraction: Araki Y (1983), Akamine Y (1986): Data abstracted from secondary source: Osakabe Y, et al. [25] Ikegami K (1998), Kaneko Y (2006), Kikuchi Y (2006), Hino H (2009), Ito T (2009), Kawaura T (2009): Data abstracted from secondary source: Hayashi M, et al. [26] Original reports are cited whenever accessible. WBC, white blood cell count; RBC, red blood cell count; Hb, hemoglobin; Hct, hematocrit; Plt, platelet count; PSL, prednisolone; mPSL, methylprednisolone; ECMO, extracorporeal membrane oxygenation; NA, not available

Author (years)	Number	Age	Sex	Severity	Occupation	Steroid use	Mechanical ventilation	Outcome/prognosis
Matsuzaki Y (1978) [17]	1	54	M	severe	boiler maintenance	Yes	No	survived
Horvath (1978) [14]	2	63	M	NA	non-smoking daily farmer	Yes	No	survived
		19	M	severe	employee of munitions	Yes	No	survived
Araki Y (1983) [*]	1	48	M	mild	automobile mechanic	Yes	No	survived
Tamura N (1985) [18]	1	50	M	mild	iron works	No	No	survived
Akamine Y (1986) [*]	1	52	M	severe	metal refining	Yes	Yes	survived
Kitahara (1988) [19]	1	33	M	severe	metal plating factory	Yes	No	survived
Iwami F (1988) [20]	1	20	M	severe	electric welder	Yes	No	survived
Shiramine K (1989) [21]	1	52	M	NA	daily farmer	Yes	Yes	survived
Hajela (1990) [3]	3	36	M	severe	pulp-mill worker	No	Yes	died
		44	M	severe	pulp-mill worker	No	Yes	died
		21	M	severe	pulp-mill worker	No	No	died
Zwemer (1992) [22]	20	NA	NA	10 severe	NA	Yes(7 cases)/No(3 cases)	NA	4 died/6 survived
									NA	NA	10 mild	NA	Yes(6 cases)/No(4 cases)	NA	10 survived
		Suzuki K (1993) [23]	6	41	M	severe	ship repairing	Yes	No	survived
60	M			severe	ship repairing	Yes	No	survived
48	M			NA	ship repairing	NA	No	survived
53	M			NA	ship repairing	NA	No	survived
39	M			NA	ship repairing	NA	No	survived
25	M			mild	ship repairing	NA	No	survived
Shimatsu Y (1996) [24]	4	47	M	severe	iron works	Yes	No	survived
		53	M	severe	iron works	Yes	No	survived
		57	M	mild	iron works	No	No	survived
		40	M	mild	iron works	No	No	survived
Bur (1997) [11]	1	56	M	severe	cleaning staff	Yes	Yes	died
Ikegami K (1998) [*]	2	NA	NA	mild	metal plating factory	NA	Yes	survived
		NA	NA	NA	metal plating factory	NA	Yes	survived
Osakabe Y (2000) [25]	1	64	M	severe	metal plating factory	Yes	Yes	survived
Kaneko Y (2006) [*]	3	65	M	severe	pump demolition work	Yes	Yes	survived
		51	M	severe	pump demolition work	Yes	No	survived
		58	M	mild	pump demolition work	Yes	No	survived
Kikuchi Y (2006) [*]	1	62	M	severe	diamond factory work	No	Yes	survived
Tanaka (2007) [10]	3	65	M	severe	factory work	Yes	Yes	survived
		52	M	severe	factory work	Yes	No	survived
		37	F	severe	factory work	Yes	No	survived
Hino H (2009) [*]	1	42	M	NA	metalworking industry	Yes	Yes	survived
Ito T (2009) [*]	1	68	M	NA	metal plating work	Yes	No	survived
Kawaura T (2009) [*]	1	21	M	NA	shipbuilding industry	Yes	Yes	survived
Jayalakshmi (2009) [8]	3	30	M	severe	cleaning staff	Yes	Yes	survived
		35	M	mild	NA	Yes	Yes	survived
		28	M	mild	NA	Yes	Yes	survived
Murphy (2010) [4]	1	66	M	severe	tank cleaning	Yes	NA	died
Lee (2014) [5]	1	50	M	mild	storekeeper	Yes	No	survived
Hayashi M (2014) [26]	1	43	M	severe	metal plating factory	No	No	survived
Kido Y (2017) [12]	1	50	M	severe	electroless nickel plating	Yes	No	survived
Present case (2018)	1	42	M	mild	chemical factory	No	No	survived

In total, 64 cases of NOx inhalation injury have been reported, with a mean age of 46.3 years (based on 42 cases with available demographic data). Severe cases, defined as those with PaO₂ <60 mmHg on room air or requiring supplemental oxygen, accounted for 34 (53.1%), whereas 21 (32.8%) were mild and 9 (14.1%) were unspecified. Overall, 55 patients (85.9%) survived and 9 (14.1%) died.

Our patient thus represents a rare category of steroid-free survival. Importantly, most previously reported survivors received corticosteroid therapy, whereas our case demonstrated full recovery without it, reinforcing the potential for conservative management in carefully monitored mild cases. Compared with previously reported severe or fatal cases, our patient presented with a milder course characterized by preserved oxygenation, radiologic-pathologic correlation, and recovery without corticosteroid therapy. This contrast underscores the clinical spectrum of NOx injury and highlights the importance of individualized management strategies.

More recent reports underscore the broad severity spectrum: dose-dependent severe cases after NO₂ explosion [9], delayed onset after occupational exposure [27], histologically confirmed secondary organizing pneumonia [28], and VV-ECMO-supported survival in severe ARDS [9,11,29]. Additional case reports have described severe lung injury and acute respiratory distress syndrome following nitric acid fume exposure [7,8]. These highlight both the life-threatening potential and the variability of NOx injury. Our case lies at the mild end of this spectrum, emphasizing the need for individualized management.

While steroids are frequently recommended, their optimal regimen remains uncertain. Some authors suggest courses of ≥4 weeks [12], yet mild cases may improve spontaneously. Lee described late-onset bronchiolitis obliterans after nitric acid exposure [5], though whether early steroids would have prevented this remains unclear. Our case suggests that in carefully monitored mild cases with preserved oxygenation, conservative management without steroids may be a reasonable option.

Imaging-pathology correlation is also noteworthy. Tanaka reported centrilobular ground-glass nodules, interlobular septal thickening, and absence of lymphadenopathy as typical HRCT features [10]. Our patient demonstrated similar CT findings, with histology showing centriacinar-dominant mural alveolitis with fibrin, closely matching radiology. Such concordance in a mild case is rarely documented and enhances understanding of NOx pathology.

## Conclusions

This case illustrates mild NOx inhalation injury following an industrial explosion, with radiologic and pathologic concordance of centriacinar-dominant mural alveolitis. It is among the very few steroid-free survivors reported. Clinically, this underscores that mild cases may resolve spontaneously, yet vigilance is essential due to potential delayed complications such as bronchiolitis obliterans and pulmonary fibrosis. Long-term follow-up is indispensable.
